# Factors associated with the presence of headache in hospitalized COVID-19 patients and impact on prognosis: a retrospective cohort study

**DOI:** 10.1186/s10194-020-01165-8

**Published:** 2020-07-29

**Authors:** Javier Trigo, David García-Azorín, Álvaro Planchuelo-Gómez, Enrique Martínez-Pías, Blanca Talavera, Isabel Hernández-Pérez, Gonzalo Valle-Peñacoba, Paula Simón-Campo, Mercedes de Lera, Alba Chavarría-Miranda, Cristina López-Sanz, María Gutiérrez-Sánchez, Elena Martínez-Velasco, María Pedraza, Álvaro Sierra, Beatriz Gómez-Vicente, Juan Francisco Arenillas, Ángel L. Guerrero

**Affiliations:** 1 Department of Neurology, Hospital Clínico Universitario de Valladolid, Avenida Ramón y Cajal 3, 47003 Valladolid, Spain; 2 Imaging Processing Laboratory, Universidad de Valladolid, Valladolid, Spain; 3 Department of Medicine, University of Valladolid, Valladolid, Spain; 4 Neurovascular Research Laboratory. Instituto de Biología y Genética Molecular, Universidad de Valladolid – Consejo Superior de Investigaciones Científicas, Madrid, Spain

**Keywords:** COVID-19, Nervous system diseases, Headache disorders, secondary, Mortality, laboratory parameters

## Abstract

**Introduction:**

Headache is one of the most frequent neurologic manifestations in COVID-19. We aimed to analyze which symptoms and laboratory abnormalities were associated with the presence of headache and to evaluate if patients with headache had a higher adjusted in-hospital risk of mortality.

**Methods:**

Retrospective cohort study. We included all consecutive patients admitted to the Hospital with confirmed SARS-CoV-2 infection between March 8th and April 11th, 2020. We collected demographic data, clinical variables and laboratory abnormalities. We used multivariate regression analysis.

**Results:**

During the study period, 576 patients were included, aged 67.2 (SD: 14.7), and 250/576 (43.3%) being female. Presence of headache was described by 137 (23.7%) patients. The all-cause in-hospital mortality rate was 127/576 (20.0%). In the multivariate analysis, patients with headache had a lower risk of mortality (OR: 0.39, 95% CI: 0.17–0.88, *p* = 0.007). After adjusting for multiple comparisons in a multivariate analysis, variables that were independently associated with a higher odds of having headache in COVID-19 patients were anosmia, myalgia, female sex and fever; variables that were associated with a lower odds of having headache were younger age, lower score on modified Rankin scale, and, regarding laboratory variables on admission, increased C-reactive protein, abnormal platelet values, lymphopenia and increased D-dimer.

**Conclusion:**

Headache is a frequent symptom in COVID-19 patients and its presence is an independent predictor of lower risk of mortality in COVID-19 hospitalized patients.

## Background

Coronavirus disease 2019 (COVID-19) is caused by severe acute respiratory syndrome coronavirus 2 (SARS-CoV-2) [1]. The severity of symptomatic cases varies from mild to critical. In the case of Spain, analyzing data from more than 250,000 infected patients, 38.4% of patients needed hospitalization, 3.9% were admitted to the Intensive Care Unit (ICU) and 8.2% expired [2]. Several factors may affect the prognosis, including male sex, older age, hypertension, diabetes, smoking, cardiovascular disorders, chronic neurological disorders, and pulmonary diseases, among others [3, 4]. Regarding clinical manifestations, fever and dyspnea have been associated with severity and mortality of COVID-19 as well [3].

Headache is one of the most frequent neurologic manifestations in COVID-19. The reported frequency of headache in a recent meta-analysis was 12% of patients [5], ranging from 6.5% [6] to 70.3% [7] depending on the studies, with most of the studies reporting a frequency within 10–30% of patients [8, 9]. Headache might be caused by the release of cytokines, endothelial damage and macrophage activation; or it could be related to the inflammation of lymphatic structures, as the lymphatic organs located in the naso- and oropharynx around the Waldeyer’s ring [10, 11]. The real causes of headache in COVID-19 are still uncertain. Whether the presence of headache reflects a more severe course of COVID-19, or if it is associated with a better prognosis because of a more efficient host response against the virus is also unclear.

In the present study, we aimed to evaluate whether prognosis of hospitalized patients with COVID-19, evaluated by in-hospital all-cause mortality rate, differed in patients with headache compared to those without headache. As a secondary objective, we assessed which variables were associated with the presence of headache in COVID-19 patients. We hypothesized that the presence of headache might be related to a different COVID-19 presentation in terms of severity and inflammatory response.

## Methods

We performed an observational analytic study with a retrospective cohort design. The study was done in a tertiary academic public hospital (Clinic University Hospital of Valladolid, Valladolid, Spain) with a reference population of 280,000 patients. The study was performed according to the strengthening the reporting of observational studies in epidemiology (STROBE) statement [12]. The study was designed and data were analyzed by the authors. All co-authors contributed to the collection, analysis and interpretation of data, as well as to the reviewing and approving of the final version of the manuscript.

We included all consecutive patients that were admitted to the Hospital with a confirmed SARS-CoV-2 infection diagnosis between March 8th and April 11th, 2020. The diagnosis was set, according to the World Health Organization (WHO) protocols [13], by real-time reverse transcriptase polymerase -chain reaction (RT-PCR) assay (LightMix Modular SARS-CoV (COVID19) E-gene and LightMix Modular SARS-CoV-2 RdRP, Roche Diagnostics) from oropharyngeal swab, sputum sample, or lower respiratory tract sample; or by the presence of anti-SARS-CoV-2 IgM + IgA antibodies (COVID-19 ELISA IgM + IgA, Vircell, S.L. Granada, Spain) in patients with clinical symptoms. Patients were excluded if the hospitalization was not done from the emergency department (ED) or if the patients’ records were not accessible.

### Data sources

Data were collected from electronic medical records. The information was gathered from primary care records, emergency room records, and discharge reports. Data were extracted between April 21st and May 1st, and the minimum follow-up time after admission was 20 days (ranging from 20 to 61 days) and the last patients were contacted on May 25th. Details of the study have been described elsewhere [4]. In those patients in which the presence of headache was not described, a trained neurologist contacted the patient or their relatives by phone and specifically inquired about it. All patients were evaluated after the resolution of all symptoms COVID-19 or after 20 days of COVID-19 symptoms. The detailed description of the headache screening process is available in supplementary materials. We analyzed the presence of any new onset headache developed in temporal relation to the onset of the viral infection, or any headache described as different to the pre-existing headache. During the hospitalization, patients were treated according to the national standard of care [14, 15].

### Study objectives

The primary objective was to evaluate if hospitalized COVID-19 patients with headache had a higher risk of all-cause in-hospital mortality compared to those without headache. The secondary objective was to analyze which variables were independently associated with the presence of headache in COVID-19 patients.

### Variables

Baseline variables included demographic data, presence of comorbidities, and details about treatment for any chronic diseases. Demographic data included age, sex and baseline performance status according to the modified Rankin scale (mRS) [16]. The evaluated comorbidities included hypertension, diabetes, smoking habits, cardiovascular diseases, pulmonary diseases, cancer, an immunocompromised state, and chronic neurological diseases [4], and we also assessed whether patients had a prior history of headache. We analyzed whether patients were previously treated with angiotensin-converting enzyme *inhibitors* (ACEis), angiotensin *II receptor blockers (ARBs)*, non-steroidal anti-inflammatory drugs (NSAIDs), corticosteroids, or other neuropharmacological therapies.

We evaluated the presence of COVID-19 general symptoms, including anosmia, arthralgia, asthenia, chest pain, cough, cutaneous rash, diarrhea, dyspnea, expectoration, fever, lightheadedness, myalgia, odynophagia, rhinorrhea, syncope, vomiting and weakness. Regarding laboratory parameters, we described both the first determination and the worst value during the hospitalization period, and we also analyzed the frequency of abnormal values. The studied parameters were leukocytes (cell count × 10^9^/L, reference value (RV): 4–10), lymphocytes (count × 10^9^/L, RV: 0.9–5.2), platelets (count × 10^9^/L, RV: 150–400), hemoglobin (g/dL, RV: 12–16), international normalized ratio (INR; RV: 0.8–1.3), D-dimer (ng/dL, RV: < 500), lactate dehydrogenase (LDH; U/L, RV: 135–250), creatine kinase (U/L, RV: 20–170), glomerular filtration rate (GFR) corrected by body area (ml/min/1.73m^2^, RV > 90), C-reactive protein (CRP; mg/L, RV: 1–5), procalcitonin (PCT; ng/mL, RV < 5), interleukin-6 (IL-6; pg/mL, RV < 5.9), and ferritin (ng/mL, RV: 15–150). We described whether chest imaging was abnormal by using either X-ray or computerized tomography scan. We analyzed the all-cause in-hospital mortality.

### Ethics

The study protocol was approved by the Institutional Review Board of the East Valladolid health area (PI-20-1751). Because of the risk of contagion, the retrospective nature of the study and the urgent need to obtain data, written informed consent was waived. However, all patients were informed about the nature of the study and were invited to participate when contacted by phone. In those patients that were personally interviewed, written informed consent was obtained. Personal information about patients was codified before entry into the database, and the identities of patients were anonymous. The study was done in accordance with the principles of the Declaration of Helsinki [17].

### Statistical analysis

We describe qualitative and ordinal variables as frequencies and percentages, respectively, and quantitative continuous variables as means and standard deviations (SD) if the distribution was normal or medians and interquartile ranges (IQR) otherwise. We assessed normality by using the Kolmogorov-Smirnov test. We used two-tailed chi-square tests or Fisher’s exact tests for hypothesis testing of qualitative variables, and an independent samples Student’s t-test or a median test for hypothesis testing of quantitative, continuous variables if the distribution was normal or not normal, respectively. The level of significance was set to 0.05, adjusting for multiple comparisons by using the Bonferroni method. We managed missing data by using complete case analysis. For the primary objective, we employed a univariate logistic regression analysis to determine which variables were associated with higher odds of mortality, including the presence of headache. Those variables with *p*-values less than or equal to 0.2 were included in a subsequent multivariate logistic regression analysis. We present the odds ratio (OR) and the 95% confidence interval (CI). To analyze which variables were associated with the presence of headache, we employed a univariate regression analysis. We were more stringent due to the high number of studied variables, so we further studied only those variables that had a *p*-value less than or equal to 0.1 in the univariate regression model. The multivariate logistic regression analysis was performed following the Wald procedure and a backwards strategy. We adjusted for multiple comparisons by using the False Discovery Rate (FDR) with Benjamini-Hochberg procedure [18]. Statistical significance was achieved if the *p*-value was < 0.05 after correcting for multiple comparisons. We analyzed multicollinearity by using the variance inflation factor (VIF). We considered multicollinearity as critical when VIF was > 5. We did not estimate the sample size a priori and the analysis proceeded on the available data. The analysis of the study was preplanned. Statistical analysis was performed with SPSS v.26 (IBM Corp. Armonk, NY).

## Results

During the study period, 580 consecutive patients fulfilled inclusion criteria. Three patients were excluded because they were not admitted from the ED and one because there was no available information. Diagnosis was confirmed by RT-PCR in 546/576 (94.8%) of cases and/or serology in 175/576 (30.4%). The mean age was 67.2 (SD: 14.7), and 250/576 (43.3%) were female.

Presence of headache was described by 137 (23.7%) patients. The headache was already present at the moment of the ED consultation in 124/137 (90.5%) patients. The headache was the first symptom in 27/104 (26.0%) patients and was present within 24 h in 40/104 (38.5%) patients, within 48 h in 65/104 (62.5%) patients and within 72 h in 77/104 (74.0%) patients. Female sex and prior history of headache were more frequent in patients with headache, while mean Rankin scale, and frequency of hypertension, smoking habits, cardiological disorders and chronic neurological disorders were more frequent in patients without headache after adjusting for multiple comparisons (*p* = 0.0029). Demographic data, comorbidities, and data about the chronic treatment of the full sample and the group of patients with and without headache are available in Table 1. Prior episodes of headache associated with systemic viral infections were described by 50/106 (47.2%) of patients with headache, and the present episode was described to be similar to the former in 23/50 (46.0%) of the cases. During the hospitalization, 55 (40.1%) patients with headache received systemic steroids compared to 245 (55.9%) patients without headache (*p* = 0.002). All-cause in-hospital mortality rate was 127/576 (20.0%) in the whole sample and 8/137 (5.8%) in headache patients.

**Table 1:** Frequency of baseline variables the entire sample and comparison between patients with and without headache

Variable	All patients (*n* = 576)	Headache (*n* = 137)	No headache (*n* = 439)	Adjusted *p*-value
Female sex	250 (43.3%)	80 (58.4%)	170 (38.7%)	**< 0.001**
Age (years)	67.2 (SD: 14.7)	59.25 (SD: 12.4)	69.6 (SD: 14.5)	**< 0.001**
Modified Rankin scale (mean)	0.61 (SD: 1.12)	0.15 (SD: 0.5)	0.75 (SD: 1.2)	**< 0.001**
Hypertension	300 (52.1%)	52 (38%)	248 (56.5%)	**< 0.001**
Diabetes	113 (19.6%)	20 (14.6%)	93 (21.2%)	0.090
Smoking	118 (20.5%)	20 (14.6%)	98 (22.3%)	0.050
Cardiological disorders	154 (26.7%)	19 (13.9%)	135 (30.8%)	**< 0.001**
Pulmonary disorders	145 (25.2%)	33 (24.1%)	112 (25.5%)	0.737
Cancer	94 (16.3%)	18 (13.1%)	76 (17.3%)	0.249
Immuno-suppression	24 (4.2%)	4 (2.9%)	20 (4.6%)	0.409
Chronic neurological disorders	105 (18.3%)	13 (9.5%)	92 (2%)	**0.002**
Prior history of headache	32 (5.6%)	15 (10.9%)	17 (3.9%)	**0.002**
ACEis /ARBs	215 (37.3%)	42 (30.7%)	173 (39.4%)	0.064
NSAIDs	75 (13%)	12 (8.8%)	66 (14.4%)	0.090
Corticosteroids	35 (6.1%)	7 (5.1%)	28 (6.4%)	0.587
Neuropharmacological treatment	203 (35.2%)	42 (30.7%)	161 (36.7%)	0.198
Time symptoms-ED (days)	7.34 (SD: 6.16)	8.51 (SD: 5.90)	6.98 (SD: 6.21)	0.011

*ACEis* angiotensin-converting enzyme *inhibitors, ARBs* angiotensin *II receptor blockers, NSAIDs* non-steroidal anti-inflammatory drugs, *ED* Emergency department. In bold, variables that were statistically significant after adjusting for multiple comparisons (threshold *p* < 0.0029)

### Primary objective: mortality

In the univariate regression analysis, baseline disability, age, hypertension, diabetes, smoking habit, cardiological disorders, cancer, and chronic neurological disorders were associated with higher odds of death, while presence of headache and time between onset of symptoms and ED visit were associated with a lower odds of death. In the multivariate regression analysis, baseline disability (OR: 4.10, 95% CI: 2.09–8.05; *p*-value: < 0.001, FDR-corrected *p*-value: < 0.001), age (OR: 1.06, 95% CI 1.03–1.08; *p*-value: < 0.001, FDR-corrected *p*-value: < 0.001) and headache (OR: 0.39, 95% CI: 0.17–0.88; *p*-value: 0.028, FDR-corrected *p*-value: 0.007) remained statistically significant. Full results of the regression analysis are presented in Table 2.

**Table 2:** Predictors of mortality: univariate and multivariate logistic regression analysis

	Type of analysis	OR	95% CI	*p*-value
mRS > 2	Univariate	11.371	6.376–20.278	**< 0.001**
	Multivariate	3.714	1.880–7.338	**< 0.001**
Age	Univariate	1.090	1.069–1.112	**< 0.001**
	Multivariate	1.058	1.055–1.031	**< 0.001**
Female sex	Univariate	0.682	0.454–1.024	0.065
	Multivariate	0.703	0.413–1.196	0.194
Hypertension	Univariate	3.534	2.272–5.495	**< 0.001**
	Multivariate	1.379	0.802–2.370	0.245
Diabetes	Univariate	2.129	1.353–3.351	**0.001**
	Multivariate	1281	0.739–2.221	0.377
Smoking	Univariate	1.589	1.004–2.514	**0.048**
	Multivariate	1.907	0.997–3.275	0.051
Cardiological disorders	Univariate	2.955	1.950–4.478	**< 0.001**
	Multivariate	1.263	0.760–2.100	0.368
Pulmonary disorders	Univariate	1.434	0.928–2.217	0.105
	Multivariate	0.898	0.517–1.559	0.702
Cancer	Univariate	1.641	1.001–2.690	**0.049**
	Multivariate	1.254	0.694–2.266	0.453
Chronic neurological disorders	Univariate	3.961	2.516–6.234	**< 0.001**
	Multivariate	1.659	0.947–2.906	0.077
Immunosuppression	Univariate	1.295	0.405–4.138	0.663
Headache	Univariate	0.167	0.079–0.351	**< 0.001**
	Multivariate	0.399	0.175–0.906	**0.028**
Time between onset of symptoms and ED visit	Univariate	0.908	0.870–0.947	**< 0.001**
	Multivariate	0.962	0.925–1.000	0.050

*OR* Odds Ratio, *CI* Confidence-interval, *mRS* Modified Rankin scale, *ED* Emergency department

### Associated symptoms and laboratory abnormalities

Anosmia, arthralgia, cough, lightheadedness, and myalgia were statistically more frequent in patients with headache after adjusting for multiple comparisons (*p* = 0.0029). The frequencies of associated symptoms in the full sample and comparisons between patients with and without headache are presented in Table 3. When comparing patients with and without headache, we observed significant differences in median values of laboratory parameters on admission for CRP, PCT, and D-dimer (*p* < 0.0025 for all after adjusting for multiple comparisons). Of the worst experienced laboratory values during hospitalization, median values were significantly different for leukocytes, lymphocytes, LDH, CRP, PCT, ferritin, IL-6, D-dimer and INR for those with and without headache. Table 4 shows the median values of laboratory parameters.

**Table 3:** Frequency of associated symptoms in the entire sample and the comparison between patients with and without headache

Variable	All patients (*n* = 576)	Headache (*n* = 137)	No Headache (*n* = 439)	Adjusted *p*-value
Anosmia	146 (25.3%)	64 (46.7%)	82 (18.7%)	**< 0.001**
Arthralgia	35 (6.1%)	18 (13.1%)	17 (3.9%)	**< 0.001**
Asthenia	242 (42.1%)	70 (51.1%)	172 (39.2%)	0.014
Chest pain	99 (17.2%)	33 (24.1%)	66 (15.1%)	0.015
Cough	403 (70.2%)	113 (82.5%)	290 (66.2%)	**< 0.001**
Cutaneous rash	11 (1.9%)	4 (2.9%)	7 (1.6%)	0.322
Diarrhea	192 (33.4%)	54 (39.4%)	138 (31.4%)	0.084
Dyspnea	292 (50.8%)	73 (53.3%)	219 (49.9%)	0.487
Expectoration	90 (15.7%)	24 (17.5%)	66 (15.0%)	0.484
Fever	462 (80.3%)	121 (88.3%)	341 (77.7%)	0.006
Lightheadedness	60 (10.4%)	19 (13.9%)	41 (9.3%)	**0.001**
Myalgia	139 (24.1%)	55 (40.1%)	85 (19.1%)	< 0.001
Odynophagia	60 (10.4%)	22 (16.1%)	38 (9.7%)	0.892
Rhinorrhea	12 (2.1%)	2 (1.5%)	10 (2.3%)	0.558
Syncope	43 (7.5%)	9 (6.6%)	34 (7.7%)	0.648
Vomiting	47 (8.2%)	11 (8%)	136 (8.2%)	0.949
Weakness	90 (15.7%)	26 (19.0%)	64 (14.6%)	0.216

In bold, variables that were statistically significant after adjusting for multiple comparisons (threshold *p* < 0.0029)

**Table 4:** Median values of laboratory parameters in the whole sample and the comparison between patients with and without headache

Variable	All patients (*n* = 576)	Headache (*n* = 137)	No headache (*n* = 439)	*p*-value
Leukocytes at admission	6710 (4945–8885.00)	6290.00 (4682.50–8257.50)	6785.00 (5137.50–9302.50)	0.202
Worst leukocyte count	9425.00 (4442.50–14,182.50)	6245.00 (3875.00–11,422.50)	9975.00 (4722.50–14,625.00)	**< 0.001**
Lymphocytes at admission	990.00 (710.00–1387.50)	1117.00 (850.00–1495.00)	940.00 (680.00–1350.00)	0.003
Worst lymphocyte count	690.00 (430.00–1050.00)	910.00 (575.00–1220.00)	650.00 (410.00–990.00)	**0.001**
Platelets at admission	195,000.00 (156,000.00–257,000.00)	197,500.00 (170,500.00–240,750.00)	194,000.00 (149,000.00–264,000.00)	0.677
Worst platelet count	192,500.00 (137,000.00–341,250.00)	206,000.00 (163,000.00–384,500.00)	188,000.00 (134,000.00–333,500.00)	0.111
LDH at admission	285.00 (225.00–372.00)	263.00 (215.00–341.00)	292.50 (227.00–381.50)	0.019
Worst LDH value	285.00 (225.00–372.00)	301.50 (237.00–411.75)	365.00 (268.50–487.00)	**< 0.001**
CK at admission	83.00 (49.00–372.00)	72.00 (49.5–110)	86.00 (47.00–166.00)	0.605
Worst CK value	89.00 (53.25–179.75)	79.00 (49.00–136.00)	94.00 (55.50–192.00)	0.026
CRP at admission	65.90 (23.37–122.15)	40.55 (14.15–102.26)	73.40 (28.55–130.05)	**0.002**
Worst CRP value	105.00 (51.25–198.02)	67.90 (21.70–164.40)	113.40 (58.45–208.62)	**< 0.001**
PCT at admission	0.10 (0.06–0.26)	0.07 (0.04–0.14)	0.11 (0.07–0.30)	**0.001**
Worst PCT value	0.13 (0.06–0.43)	0.07 (0.04–0.18)	0.15 (0.08–0.54)	**< 0.001**
Worst ferritin value	979.00 (444.40–1819.00)	615.00 (306.80–1449.70)	1094.80 (534.75–1945.47)	**< 0.001**
Worst IL-6 value	27.80 (12.40–61.87)	17.95 (9.76–44.15)	33.50 (13.32–67.30)	**0.001**
D-Dimer at admission	761.50 (462.75–1369.00)	550.00 (380–864)	855.50 (515–50-1570.00)	**0.001**
Worst D-Dimer value	1300.00 (674.75–3231.75)	810.00 (504.50 (1458.50)	1534.00 (795.00–3580.00)	**< 0.001**
INR at admission	1.18 (1.10–1.29)	1.15 (1.08–1.23)	1.19 (1.11–1.32)	0.003
Worst INR value	1.25 (1.15–1.40)	1.19 (1.11–1.30)	1.26 (1.17–1.45)	**< 0.001**

*LDH* Lactate dehydrogenase, *CK* Creatine Kinase, *CRP* C-reactive protein, *PCT* Procalcitonin, *IL-6* interleukin 6, *INR* International normalized ratio. In bold, variables that were statistically significant after adjusting for multiple comparisons (threshold *p* < 0.0025)

### Variables associated with the presence of headache

**Fig. 1:**
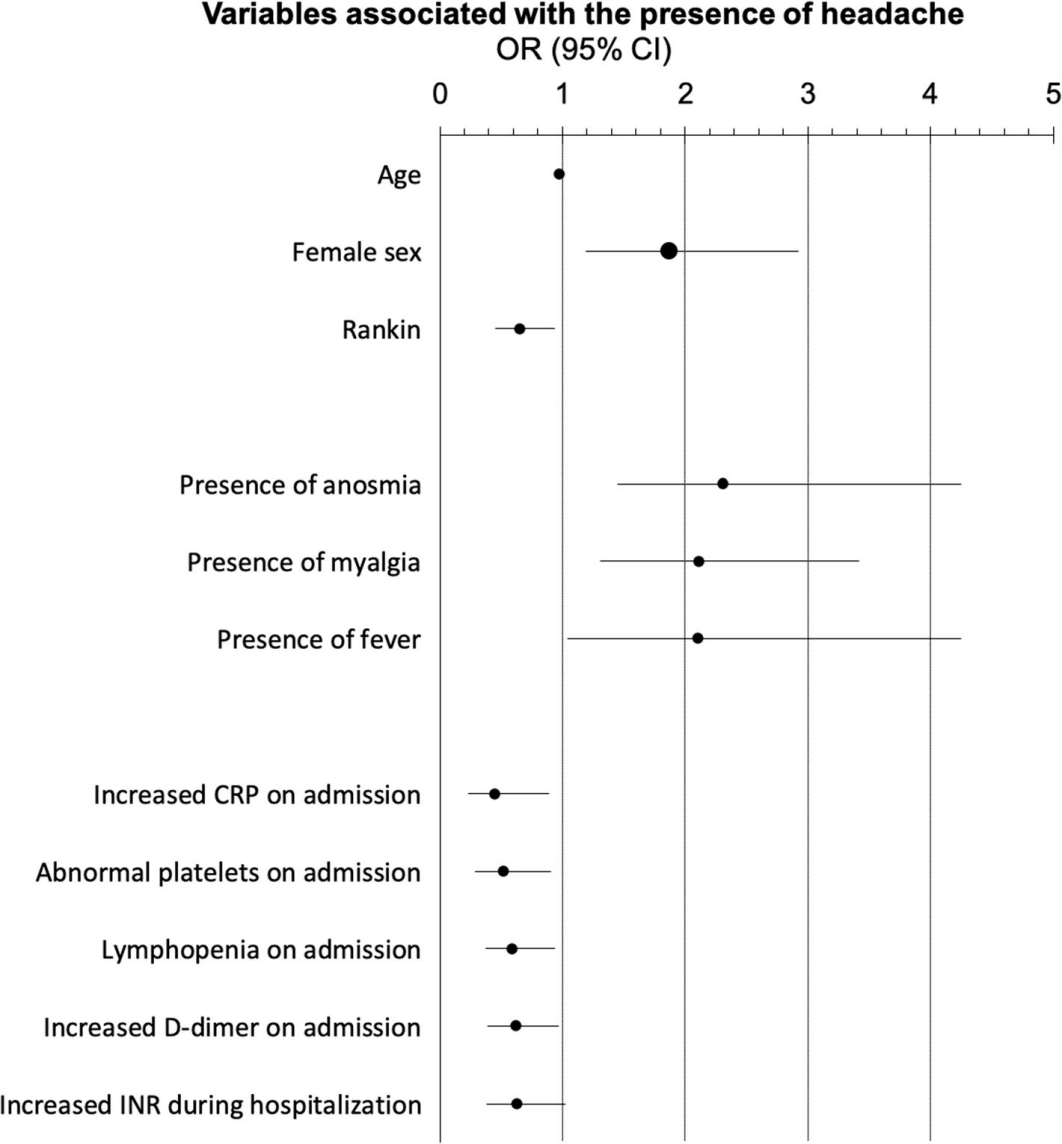
Odds ratio and 95% confidence interval of variables associated with the presence of headache

In the univariate regression analysis, 56 different variables were included in the model, of which 39 had a *p*-value < 0.1 (supplementary Table 1). We included those 39 variables in a multivariate regression analysis, and 11 variables were statistically significant. After FDR adjustments, 10 variables remained statistically significant. The variables that were independently associated with a higher odds of headache in hospitalized COVID-19 patients were anosmia, myalgia, female sex, and fever; while the variables that were independently associated with a lower odds of having headache were age, modified Rankin scale, increased CRP on admission, abnormal platelet value on admission, lymphopenia on admission, and increased D-dimer on admission. The VIF was less than or equal to 1.131 in all cases. Full results of the multivariate regression analysis are shown in Table 5 and Fig. 1.

**Table 5:** Multivariate regression analysis of variables that were associated with the presence of headache

Variable	OR	95% CI	*p*-value	Corrected *p*-value (Benjamini-Hochberg)
Presence of anosmia	2.306	1.449–4.247	< 0.001	**0.001**
Presence of myalgia	2.114	1.308–3.418	0.002	**0.011**
Age	0.976	0.959–0.993	0.005	**0.018**
Female sex	1.861	1.186–2.921	0.007	**0.019**
Rankin	0.654	0.455–0.940	0.022	**0.048**
Increased CRP on admission	0.451	0.227–0.897	0.023	**0.042**
Abnormal platelets on admission	0.516	0.291–0.912	0.023	**0.036**
Lymphopenia on admission	0.59	0.368–0.945	0.028	**0.031**
Presence of fever	2.105	1.043–4.247	0.038	**0.046**
Increased D-dimer on admission	0.616	0.388–0.978	0.04	**0.044**
Increased INR during hospitalization	0.627	0.384–1.024	0.062	0.062

In bold, variables that were statistically significant after adjusting for multiple comparisons (Benjamini-Hochberg procedure)

## Discussion

In this study we analyzed which variables were associated with the presence of headache in COVID-19 patients and whether patients with headache had a worse prognosis, assessed by the risk of all-cause, in-hospital mortality. For this, we studied a large cohort of hospitalized patients with confirmed COVID-19, and we systematically interrogated them about the presence of headache.

SARS-CoV-2 causes respiratory symptoms in most patients. However, neurological symptoms are among the most frequent extrapulmonary symptoms [8, 9]. After an incubation period of 2–14 days [1], most patients present general symptoms for 1–3 weeks [19]. Patients with comorbidities are particularly vulnerable to the virus [4, 20], and there are several risk factors that have been associated with a worse prognosis [3, 21, 22]. The prompt diagnosis, isolation and treatment of patients is crucial [23]. Every clinician should be prepared for facing the virus, as the pandemic has extended worldwide causing hundreds of thousands of deaths and millions of cases [2].

Headache is a frequent symptom of COVID-19. It was present in almost a quarter of our sample. This frequency was higher than in other previously reported series, which described a 6–15% prevalence [5, 6, 8, 9]. On the one hand, this finding must be interpreted with caution given that we only included hospitalized patients. On the other hand, this could be explained because we interviewed every patient or relative about the presence of headache, reaching them by telephone if necessary. We also extensively reviewed primary care records, screening for the presence of headache. Further studies will be necessary to clarify the real prevalence of headache in COVID-19 patients, including outpatient series.

The main finding of our study was that headache was associated with a lower probability of death. In our sample, headache patients had a different clinical picture, as the frequency of symptoms such as anosmia, cough, myalgia and arthralgia were higher. The higher frequency of symptoms could suggest that those patients visited the ED earlier; however, in our sample, patients with headache presented to the ED later than those without headache. This could reflect that patients without headache suffer from a more severe case of COVID-19 and therefore they seek medical attention earlier [24].

Many laboratory results were also different in patients with headache compared with the rest of the sample, although these results should be interpreted cautiously [25]. Headache patients were younger, more frequently female, less disabled, and had lower frequencies of hypertension, smoking habits, cardiac disorders, and chronic neurological disorders. All of those could influence the crude results [3, 4], and therefore we did a multivariate regression analysis to assess which variables were independently associated with the presence of headache.

The baseline factors that independently increased the odds of having headache were the female sex, younger age, and lower disability level at the baseline. These variables have been associated with a lower risk of death from COVID-19 [4, 26]. However, in our study, headache was independently associated with a lower risk of death in the multivariate model.

Some symptoms were associated with higher odds of headache, such as fever, anosmia and myalgia [27]. Fever, myalgia and headache are common in other systemic viral infections [28–30]. These symptoms can be related to lymphocyte and macrophage activation, interferon secretion and cytokine release [31, 32]. An efficient innate immunity response is associated with a better COVID-19 prognosis. However, in some patients, the immune response persists and causes endothelial dysfunction, thrombosis, persistent macrophage activation, fibrinoid organizing pneumonia, acute respiratory distress syndrome, multi-organ failure and death [33].

Laboratory biomarkers reportedly associated with COVID-19 pathophysiology include lymphocytes, CRP, D-dimer [34], PCT, troponins, ferritin, and IL-6, among others [22, 35]. In our sample, most of them differed between patients with and without headache. In the multivariate analysis, patients with headache had lower odds of having increased CRP, abnormal platelet values, lymphopenia and increased D-dimer at the ED visit, and these are factors that are typically associated with the cytokine storm described in COVID-19 patients [32, 36]. In our opinion, this could partially explain the lower risk of death experienced by COVID-19 patients with headache [36]. However, it was also an unexpected finding, as we hypothesized that the presence of headache could be partially explained by those factors. We could not explain the lower levels of CRP or IL-6 due to the use of NSAIDs or steroids, as patients with headache received them less frequently than the rest of the sample. The specific causes of headache might be further studied via imaging and systematic cerebrospinal fluid evaluation.

Our findings should be interpreted with caution. In a previous prospective study that included 179 hospitalized COVID-19 patients with pneumonia, mortality was higher in patients with headache (5/21 (23.8%)) than those without headache (12/158 (7.6%)); however, it did not remain statistically significant in the multivariate regression analysis [37]. In a meta-analysis that included 19 studies (2874 patients) published between January 1, 2020 and February 21, 2020, presence of headache was not associated with a worse prognosis [38]. We propose that the impact of headache in COVID-19 prognosis should be studied in deeper detail. Compared with other symptoms, headache might have different phenotypes and each presentation might have a different meaning [39, 40]. We hypothesize that the impact of headache on prognosis might be linked to the causes of headache [41]. If the presence of headache represents a more effective immune response, then it could be related to a lower mortality. In those cases in which the headache is associated with systemic complications, the prognosis might be worse. However, in our study, most of the patients reported the headache early in the course of the disease, and therefore the precise significance of later or different headache presentations should also be analyzed.

This study has notable weaknesses. It was a single-center study, so the results may not be applicable to other centers or countries. In addition, the sample included only hospitalized patients, which could limit the generalizability of the results. Further multicentric studies with larger sample sizes and inclusion of non-hospitalized patients will be necessary to evaluate our hypothesis and to clarify whether the prevalence of headache differs between admitted and non-admitted patients, particularly in patients with prior headache history. Finally, our study was retrospective and the collected data may not be complete; despite the extensive search, the frequency of headache could be underreported due to the lack of prospective and iterative evaluation, the unavailability of some patients, and the fact that in some cases we contacted the relatives instead of the patients. Some variables may be associated with the lower age and higher frequency of female sex seen in patients with headache, so direct comparisons should be interpreted cautiously. We did not assess the impact on prognosis of the different possible headache phenotypes. We encourage other researchers to pool the data and create a larger series that could better clarify the role of prior history of headache disorders in the prognosis and phenotyping of headache in COVID-19.

## Conclusion

Headache is a frequent symptom in patients with COVID-19. The presence of headache is an independent predictor of lower risk of mortality in COVID-19 hospitalized patients. Anosmia, myalgia, fever and female sex were independent predictors of headache in hospitalized COVID-19 patients, while lower age and lower baseline disability, CRP, platelet value, lymphocyte count and D-dimer values on admission were independently associated with the presence of headache.

## Supplementary information

**Additional file 1:** Summary of the screening proccess.

## Data Availability

The datasets used and/or analysed during the current study are available from the corresponding author upon reasonable request*.*

## Abbreviations

ACEis: Angiotensin-converting enzyme *inhibitors*
*ARBs*: Angiotensin *II receptor blockers*
NSAIDs: Non-steroidal anti-inflammatory drugs
*CI*: *Confidence interval*
COVID-19: Coronavirus disease 2019
CRP: C-reactive protein
ED: Emergency department
GRR: Glomerular filtration rate
ICU: Intensive Care Unit
IL-6: Interleukin-6
INR: International normalized ratio
LDH: Lactate dehydrogenase
mRS: Modified Rankin scale
NSAIDs: Non-steroidal anti-inflammatory drugs
RV: Reference value
RT-PCR: Real-time reverse-transcription polymerase chain reaction
SARS-CoV-2: Severe acute respiratory syndrome coronavirus 2
time symptoms-ED: Time since the beginning of the symptoms to the arrival to emergency department
STROBE: Strengthening the reporting of observational studies in epidemiology
SD: Standard deviation
IQR: Inter-Quartile range
OR: Odds ratio
FDR: False Discovery Rate
VIF: Variance Inflation Factor
WHO: World Health Organization

## References

[1] Zhou M, Zhang X, Qu J (2020). Coronavirus disease 2019 (COVID-19): a clinical update. Front Med.

[2] National Epidemiologic Surveillance Network (2020). Covid-19 report number 33.

[3] Zheng Z, Peng F, Xu B, Zhao J, Liu H, Peng J (2020). Risk factors of critical & mortal COVID-19 cases: a systematic literature review and meta-analysis. J Infect.

[4] García-Azorin D, Martínez-Pías D, Trigo J, Hernández-Pérez I, Valle-Peñacoba G, Talavera B et al (2020) Neurological comorbidity is a predictor of death in Covid-19 disease: a cohort study on 576 patients. Front Neurol. 10.3389/fneur.2020.00781 10.3389/fneur.2020.00781 PMC7358573 32733373

[5] Borges do Nascimento IJ, Cacic N, Abdulazeem HM, Von Groote TC, Jayarajah U, Weerasekara I (2020). Novel Coronavirus Infection (COVID-19) in humans: a scoping review and meta-analysis. J Clin Med.

[6] Tian S, Hu N, Lou J, Chen K, Kang X, Xiang Z (2020). Characteristics of COVID-19 infection in Beijing. J Infect.

[7] Lechien JR, Chiesa-Estomba CM, Place S, Van Laethem Y, Cabaraux P, Mat Q et al (2020) Clinical and epidemiological characteristics of 1420 European patients with mild-to-moderate coronavirus disease 2019. J Int Med. 10.1111/joim.13089 10.1111/joim.13089 PMC7267446 32352202

[8] Mao L, Jin H, Wang M, Hu Y, Chen S, He Q (2020). Neurologic manifestations of hospitalized patients with coronavirus disease 2019 in Wuhan, China. JAMA Neurol.

[9] Li LQ, Huang T, Wang YQ, Wang ZP, Liang Y, LIang Y et al (2020) COVID-19 patients’ clinical characteristics, discharge rate, and fatality rate of meta-analysis. J Med Virol:1–7. 10.1002/jmv.25757 10.1002/jmv.25757 PMC7228329 32162702

[10] Masieri S, Trabattoni D, Incorvaia C, De Luca MC, Dell'Allbani I, Leo G, et al. A role for Waldeyer's ring in immunological response to alergens. Curr Med Res Opin. 2014;30(2):203–5. 10.1185/03007995.2013.855185. 10.1185/03007995.2013.855185 24127824

[11] El-Anwar MW, Elzayat S, Fouad YA. ENT manifestation in COVID-19 patients. Auris Nasus Larynx. 2020;15:S0385-8146(20):30146–2. 10.1016/j.anl.2020.06.003. 10.1016/j.anl.2020.06.003 PMC7294298 32586739

[12] Von Elm E, Altman DG, Egger M (2007). The strengthening the reporting of observational studies in epidemiology (STROBE) statement: guidelines for reporting observational studies. PLoS Med.

[13] World Health Organization. Coronavirus disease (COVID-19) technical guidance: laboratory testing for 2019-nCoV in humans (https://www.who.int/emergencies/diseases/novel-coronavirus-2019/technical-guidance/laboratory-guidance). Accessed 19 May 2020.

[14] Ministry of Health (2020). Technical documents. Hospital management of COVID-19.

[15] World Health Organization (2020). Clinical management of severe acute respiratory infection when novel coronavirus (2019- nCoV) infection is suspected: interim guidance.

[16] Quinn TJ, Dawson J, Walters MR, Lees KR (2009). Reliability of the modified Rankin scale: a systematic review. Stroke.

[17] International Conference on Harmonisation (1996). Good clinical practice: consolidate guideline. Proceedings of the International conference on harmonisation of technical requirements for registrations of pharmaceuticals for human use.

[18] Benjamini Y, Hochberg Y (1995). Controlling the false discovery rate: a practical and powerful approach to multiple testing. J R Stat Soc Ser B.

[19] Kaur N, Gupta I, Singh H, Karia R, Ashraf A, Habib A et al (2020) Epidemiological and clinical characteristics of 6635 COVID-19 patients: a pooled analysis. SN Compr Clin Med:1–5. 10.1007/s42399-020-00393-y PMC7343407 10.1007/s42399-020-00393-y PMC7343407 32838160

[20] Sanyaolu A, Okorie C, Marinkovic A, Patidar R, Younis K, Desai P et al (2020) Comorbidity and its impact on patients with COVID-19. SN Compr Clin Med:1–8. 10.1007/s42399-020-00363-4 PMCID: PMC7314621 10.1007/s42399-020-00363-4 PMC7314621 32838147

[21] Bwire GM (2020) Coronavirus: Why men are more vulnerable to Covid-19 than women? SN Compr Clin Med:1–3. 10.1007/s42399-020-00341-w PMCID: PMC7271824 10.1007/s42399-020-00341-w PMC7271824 32838138

[22] Pennica A, Conforti G, Falangone F, Martocchia A, Tafaro L, Sentimentale A, Marini V et al (2020) Clinical management of adult coronavirus infection disease 2019 (COVID-19) positive in the setting of low and medium intensity of care: a short practical review. SN Compr Clin Med:1–6. 10.1007/s42399-020-00333-w PMCID: PMC7258606 10.1007/s42399-020-00333-w PMC7258606 32838135

[23] Shen B, Chen L, Zhang L, Zhang M, Li J, Wu J et al (2020) Wuchang Fangcang Shelter Hospital: Practices, experiences, and lessons learned in controlling COVID-19. SN Compr Clin Med:1–6. 10.1007/s42399-020-00382-1 PMCID: PMC7334130 10.1007/s42399-020-00382-1 PMC7334130 32838157

[24] O’Reilly GM, Mitchell RD, Rajiv P, Wu J, Brennecke H, Brichko L, Noonan MP et al (2020) Epidemiology and clinical features of emergency department patients with suspected COVID-19: initial results from the COVID 19 emergency department quality improvement project (COVED-1). Emerg Med Australas. 10.1111/1742-6723.13540 10.1111/1742-6723.13540 32378797

[25] Tang YW, Schmitz JE, Persing DH, Stratton CW (2020). Laboratory diagnosis of COVID-19: current issues and challenges. J Clin Microbiol.

[26] Zhou F, Yu T, Du R, Fan G, Liu Y, Liu Z (2020). Clinical course and risk factors for mortality of adult inpatients with COVID-19 in Wuhan, China: a retrospective cohort study. Lancet.

[27] Vacchiano V, Riguzzi P, Volpi L, Tappatà M, Avoni P, Rizzo G et al (2020) Early neurological manifestations of hospitalized COVID-19 patients. Neurol Sci:1–3. 10.1007/S10072-020-04525-z 10.1007/s10072-020-04525-z PMC7330256 32617738

[28] Eccles R, Loose I, Jawad M, Nyman L (2003). Effects of acetylsalicylic acid on sore throat pain and other pain symptoms associated with acute upper respiratory tract infection. Pain Med.

[29] Ng S, Cowling BJ, Fang VJ, Chang KH, Ip DKM, Cheng CKY (2010). Effects of Oseltamivir treatment on duration of clinical illness and viral shedding and household transmission of influenza virus. Clin Infect Dis.

[30] Ishii T, Sasaki Y, Maeda T, Komatsu F, Suzuki T, Urita Y (2019). Clinical differentiation of infectious mononucleosis that is caused by Epstein-Barr virus or cytomegalovirus: a single-center case-control study in Japan. J Infect Chemother.

[31] Guo L, Ren L, Yang S, Xiao M, Chang D, Yang F, et al. Profiling early humoral response to diagnose novel coronavirus disease (COVID-19). Clin Infect Dis. 2020;ciaa310. 10.1093/cid/ciaa310. 10.1093/cid/ciaa310 PMC7184472 32198501

[32] Ye Q, Wang B, Mao J (2020). The pathogenesis and treatment of the `cytokine Storm' in COVID-19. J Inf Secur.

[33] Tay MZ, Poh CM, Rénia L, MacAry PA, Ng LFP (2020). The trinity of COVID-19: immunity, inflammation and intervention. Nat Rev Immunol.

[34] Zhang L, Yan X, Fan Q, Liu H, Liu X, Liu Z (2020). D-dimer levels on admission to predict in-hospital mortality in patients with Covid-19. J Thromb Haemost.

[35] Soraya GV, Ulhaq ZS (2020). Crucial laboratory parameters in COVID-19 diagnosis and prognosis: an updated meta-analysis. Med Clin.

[36] Meftahi GH, Jangravi Z, Sahraei H, Bahari Z (2020). The possible pathophysiology mechanism of cytokine storm in elderly adults with COVID-19 infection: the contribution of “inflame-aging”. Inflamm Res.

[37] Rong-Hui D, Li-Rong L, Cheng-Qing Y, Wen W, Tan-Ze C, Ming L (2020). Predictors of mortality for patients with COVID-19 pneumonia caused by SARS-CoV-2: a prospective cohort study. Eur Respir J.

[38] Rodriguez-Morales AJ, Cardona-Ospina JA, Gutiérrez-Ocampo E, Villamizar-Peña R, Holguin-Rivera Y, Escalera-Antezana P (2020). Clinical, laboratory and imaging features of COVID-19: a systematic review and meta-analysis. Travel Med Infect Dis.

[39] Belvis R (2020) Headaches during COVID-19: my clinical case and review of the literature. Headache. 10.1111/head.13841 10.1111/head.13841 PMC7273035 32413158

[40] Porta-Etessam J, Matías-Guiu JA, Gonzalez-García N, Gomez Iglesias P, Santos-Bueso E, Arriola-Villalobos P et al (2020) Spectrum of headaches associated with SARS-CoV-2 infection: study of healthcare professionals. Headache. 10.1111/head.13902 10.1111/head.13902 PMC7405125 32666513

[41] García-Azorín D, Trigo J, Talavera B, Martínez-Pías E, Sierra A, Porta-Etessam J et al (2020) Frequency and type of red flags in patients with Covid-19 and headache: a series of 104 hospitalized patients. Headache. 10.1111/head.13927 10.1111/head.13927 PMC7436570 32790215

