# Supplementary material for: Factors associated with the presence of headache in hospitalized COVID-19 patients and impact on prognosis: a retrospective cohort study
Source: J Headache Pain. 2020 Jul 29;21(1):94. doi: 10.1186/s10194-020-01165-8 (PMC7388434; doi:10.1186/s10194-020-01165-8)
Supplement: Supplementary file 1 — Additional file 1: Summary of the screening proccess. [file 10194_2020_1165_MOESM1_ESM.docx]

**Supplementary materials:**

**Factors associated with the presence of headache in hospitalized COVID-19 patients and impact on prognosis: A retrospective cohort study**

The headache screening process:

The screening of headache was done by four different filters:

1^st^: We analyzed if the presence of headache was mentioned in the Emergency Department records. In our hospital, there was a standardized checklist that included headache within the possible COVID-19 symptoms. (n=84).

2^nd^: we analyzed if the presence of headache was mentioned in the hospitalization records. (n=+8, total: 92)

3^rd^: we reviewed the primary care records, which included the pre-hospitalization and post-hospitalization period. According to the local protocol, general practitioners contacted their patients every day or every other day. (n=+7, total: 99).

4^th^: In those patients in which the presence of headache was not mentioned in none of the above mentioned, we contacted patients by personal interview (when they were still admitted) or by phone, either to them or their relatives. (477 were screened, n+38 had headache, total: 137). In case that the relatives were not sure about the presence or absence of headache, we contacted the patients again or marked the patients as “unclear”.

The number of patients that could not be reached at this stage were: 10 (1.7% of total, 2.1% of the screened patients).

We could not reach 5 patients after three attempts (n=3) or because there was not any available phone (n=2).

3 patients had cognitive impairment and had not a reliable informant.

2 patients rejected to participate.

Supplementary figure 1 summarizes the screening process:


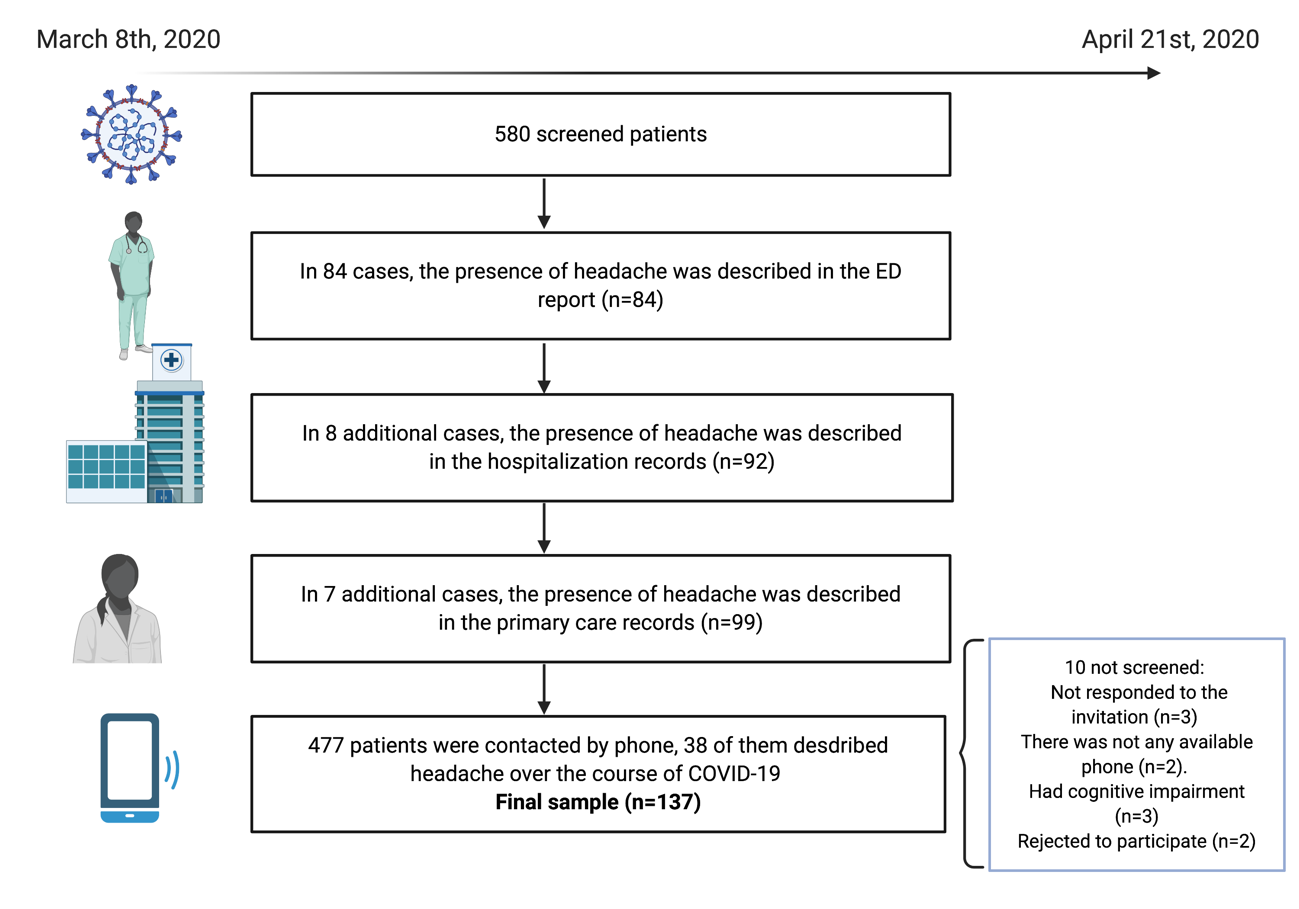


Supplementary table 1: Univariate regression analysis of variables that were associated with the presence of headache.

|  | OR | 95% CI | P-value |
| --- | --- | --- | --- |
| Female sex | 2.2221 | 1.503-3.281 | <0.001 |
| Age | 0.952 | 0.923-0.965 | <0.001 |
| Hypertension | 0.471 | 0.318-0.698 | <0.001 |
| Diabetes | 0.636 | 0.376-1.077 | 0.092 |
| Smoking habit | 0.595 | 0.352-1.005 | 0.052 |
| Prior history of cardiac disorders | 0.363 | 0.214-0.613 | <0.001 |
| Prior history of pulmonary disorders | 0.926 | 0.593-1.448 | 0.737 |
| Prior history of cáncer | 0.722 | 0.415-1.257 | 0.250 |
| Prior history of immunosuppression | 0.578 | 0.218-1.531 | 0.270 |
| Prior history of chronic neurological disorders | 0.395 | 0.214-0.732 | 0.003 |
| Prior history of headache | 3.052 | 1.481-6.289 | 0.002 |
| Modified Rankin scale | 0.432 | 0.305-0.611 | <0.001 |
| Prior use of ACE-I or ARB | 0.680 | 0.451-1.025 | 0.065 |
| Prior use of NSAIDs | 0.573 | 0.299-1.097 | 0.093 |
| Prior use of steroids | 0.790 | 0.337-1.852 | 0.588 |
| Presence of arthralgia | 3.755 | 1.877-7.511 | <0.001 |
| Presence of asthenia | 1.622 | 1.103-2.386 | 0.014 |
| Presence of weakness | 1.372 | 0.830-2.269 | 0.217 |
| Presence of diarrhea | 1.419 | 0.954-2.112 | 0.084 |
| Presence of dyspnea | 1.146 | 0.780-1.683 | 0.487 |
| Presence of chest pain | 1.788 | 1.117-2.864 | 0.016 |
| Presence of expectoration | 1.200 | 0.719-2.004 | 0.485 |
| Presence of fever | 2.173 | 1.232-3.834 | 0.007 |
| Presence of anosmia | 3.817 | 2.526-5.766 | <0.001 |
| Presence of lightheadedness | 1.563 | 0.874-2.796 | 0.132 |
| Presence of myalgia | 2.835 | 1.870-4.298 | <0.001 |
| Presence of odynophagia | 2.019 | 1.148-3.550 | 0.015 |
| Presence of rash | 1.856 | 0.535-6.438 | 0.330 |
| Presence of rhinorrhea | 0.562 | 0.636-2.936 | 0.562 |
| Presence of cough | 2.403 | 1.482-3.895 | <0.001 |
| Presence of vomiting | 0.977 | 0.483-1.976 | 0.949 |
| Abnormal leukocytes on admission | 0.495 | 0.309-0.793 | 0.003 |
| Abnormal leukocytes during hospitalization | 0.634 | 0.426-0.944 | 0.025 |
| Lymphopenia on admission | 0.475 | 0.314-0.718 | <0.001 |
| Lymphopenia during hospitalization | 0.435 | 0.295-0.643 | <0.001 |
| Anemia on admission | 0.769 | 0.469-1.262 | 0.299 |
| Anemia during hospitalization | 0.676 | 0.458-0.997 | 0.048 |
| Thrombopenia on admission | 0.450 | 0.270-0.748 | 0.002 |
| Thrombopenia during hospitalization | 0.712 | 0.482-1.051 | 0.087 |
| Increased LDH on admission | 0.616 | 0.417-0.909 | 0.015 |
| Increased LDH during hospitalization | 0.502 | 0.326-0.774 | 0.002 |
| Impaired kidney function on admission | 0.459 | 0.310-0.681 | <0.001 |
| Impaired kidney function during hospitalization | 0.449 | 0.295-0.682 | <0.001 |
| Increased INR on admission | 0.404 | 0.233-0.701 | 0.001 |
| Increased INR during hospitalization | 0.471 | 0.306-0.725 | 0.001 |
| Increased D-dimer on admission | 0.457 | 0.308-0.679 | <0.001 |
| Increased D-dimer during hospitalization | 0.461 | 0.285-0.746 | 0.002 |
| Increased CPK on admission | 0.602 | 0.275-1.317 | 0.204 |
| Increased CPK during hospitalization | 0.693 | 0.426-1.125 | 0.138 |
| Increased CRP on admission | 0.439 | 0.247-0.780 | 0.005 |
| Increased CRP during hospitalization | 0.388 | 0.166-0.905 | 0.028 |
| Increased PCT on admission | 0.462 | 0.223-0.959 | 0.038 |
| Increased PCT during hospitalization | 0.493 | 0.283-0.859 | 0.013 |
| Increased IL-6 during hospitalization | 0.998 | 0.643-1.550 | 0.993 |
| Increased ferritin during hospitalization | 0.766 | 0.422-1.393 | 0.383 |
| Abnormal chest imaging | 0.1393 | 0.517-3.750 | 0.512 |
